# Conceptualizing social risk in relation to climate change and assisted ecosystem adaptation

**DOI:** 10.1111/risa.17635

**Published:** 2024-08-23

**Authors:** Stewart Lockie, Victoria Graham, Bruce Taylor, Umberto Baresi, Kirsten Maclean, Gillian Paxton, Karen Vella

**Affiliations:** ^1^ The Cairns Institute James Cook University Cairns Australia; ^2^ School of Sociology The Australian National University Canberra Australia; ^3^ Commonwealth Scientific Industry Research Organisation Brisbane Australia; ^4^ School of Architecture and Built Environment Queensland University of Technology Brisbane Australia; ^5^ Fenner School of Environment and Society The Australian National University Canberra Australia

**Keywords:** Assisted ecosystem adaptation, climate adaptation, risk governance, social risk

## Abstract

Realizing positive social and environmental outcomes from assisted ecosystem adaptation requires the management of complex, uncertain, and ambiguous risks. Using assisted coral reef adaptation as a case study, this article presents a conceptual framework that defines social impacts as the physical and cognitive consequences for people of planned intervention and social risks as potential impacts transformed into objects of management through assessment and governance. Reflecting on its multiple uses in the literature, we consider “social risk” in relation to risks to individuals and communities, risks to First Peoples, risks to businesses or project implementation, possibilities for amplified social vulnerability, and risk perceptions. Although much of this article is devoted to bringing clarity to the different ways in which social risk manifests and to the multiple characters of risk and uncertainty, it is apparent that risk governance itself must be an inherently integrative and social process.

## 1 INTRODUCTION

Limiting the adverse social, economic, and environmental impacts of anthropogenic climate change demands both mitigation of greenhouse gas emissions to limit the scale and pace of change and adaptation to changes that are now unavoidable (IPCC, 2022). Considered evaluation of adaptation efforts is vital to ensure that costs and benefits are understood, risks are minimized, and opportunities for public dialogue and participation are enabled (Few et al., 2007; Eriksen et al., 2021).

Although climate adaptation interventions are likely to benefit communities and industries in a number of ways, it should not be assumed that benefits will be uniformly positive across space and time, or across communities and industries (Eriksen et al., 2021; Löfqvist et al., 2023). Even the potential for negative or inequitably shared outcomes may provoke conflict over adaptation programs and threaten their implementation. As decades of contestation over renewable energy projects demonstrate (Batel, 2020), failure to identify what we refer to in this article as “social risks” early may delay action to address climate threats, increase costs, and compromise possibilities for lasting and positive social change (Glucker et al., 2013).

Realizing positive social change through climate adaptation requires attention to several interrelated conceptual and methodological challenges. These include the need to account for change that is of potentially unlimited temporal and spatial reach but which can also be abrupt, localized, and discontinuous (Lockie, 2014), and the consequent need to account for uncertainty about the long‐term impacts of climate adaptation interventions that may themselves generate unanticipated, abrupt, and/or irreversible outcomes (see Schipper, 2020; Silvestrini et al., 2015; Sovacool et al., 2023). This article explores these challenges in relation to assisted ecosystem adaptation, an approach to ecological management concerned with building ecosystem resilience to current and future climate change (Lam et al., 2017) through techniques such as assisted species migration, genetic selection, and solar radiation management (Vella et al., 2021).

The context for our research is assisted adaptation research in Australia's Great Barrier Reef (GBR) which, like coral reef ecosystems worldwide, is critically threatened by climate change (IPCC, 2022). Mass coral bleaching events caused by marine heatwaves are occurring more frequently, extending over longer timeframes, and leaving shorter windows for recovery, with the 2014–2017 global bleaching event considered the worst on record. Among other responses to coral bleaching is research into strategies to support the adaptation of corals to higher temperatures (Shaver et al., 2022; Sovacool et al., 2023). The largest of these, Australia's Reef Restoration and Adaptation Program (RRAP), investigates strategies to build reef resilience through interventions designed to provide coral with short‐term protection from extreme weather events, facilitate its recovery from disturbance, and accelerate its adaptation to heat stress (McLeod et al., 2022).

Our objectives for this article are: first, to explore the challenges assisted ecosystem adaptation poses for risk assessment in general; second, to articulate a conceptual framework for social risk to inform and assess adaptation interventions; and third, to demonstrate the framework's utility in the context of assisted coral reef adaptation. The framework we articulate in this article is synthetic, drawing on existing concepts and methodologies to clarify how these can usefully be applied to risk problems characterized by complex temporal and spatial dynamics, high levels of uncertainty, and competing perspectives on ethics and feasibility.

## 2 ASSISTED ECOSYSTEM ADAPTATION

This section introduces RRAP as an exemplar of innovation in assisted ecosystem adaptation and the challenges assisted adaptation raises for risk management.

### 2.1 Case context: The RRAP

The RRAP is a multi‐institutional research partnership^1^ focused on providing reef managers with a suite of scientifically proven, ecologically effective, socially acceptable, technically feasible, and economically viable options to intervene at scale on the GBR and other reefs to enhance their resilience and accelerate adaptation to climate change. Following a two‐year pre‐feasibility assessment of 160 possible interventions (Bay et al., 2019), RRAP commenced detailed research in 2020 on:

- Solar radiation management (SRM) interventions, including seawater fogging and marine cloud brightening—the former to provide short‐term protection of high‐priority sites during marine heatwaves and the latter to reduce ocean temperatures throughout the GBR lagoon (Butcherine et al., 2023; Harrison, 2024). In contrast with prospective SRM technologies based on the injection of sulfate particles into the stratosphere (Irvine et al., 2016), options under investigation by RRAP use atomized seawater to generate low‐lying fog and to increase the reflectivity of clouds, respectively.
- Enhanced reef restoration interventions, including techniques to increase the recruitment of juvenile corals to damaged reefs by harvesting and moving larval slicks and techniques to increase the efficiency of coral aquaculture and outplanting (Lippmann et al., 2023; McLeod et al., 2022; Randall et al., 2021). Research is also under way to understand the effect of coral rubble on damaged reefs and the potential value of intervening to provide a more stable substrate for coral recovery (Kenyon et al., 2023).
- Coral adaptation interventions, including the identification of corals with natural heat resilience traits, selective breeding for heat tolerance within and between coral populations, preconditioning of juvenile corals to heat stress, and introduction of heat‐evolved strains of algae on which corals rely for photosynthetic production of energy (McLeod et al., 2022; Scharfenstein et al., 2023; Selmoni et al., 2024). Cryopreservation is being used beyond its usual role in genetic conservation to increase the efficiency of coral breeding and aquaculture (Daly et al., 2022).
- Fundamental ecosystem and evolutionary processes implicated in coral reef recovery and adaptation and the modeling and decision‐support systems needed to utilize this knowledge for effective intervention (Anthony et al., 2020; Sivapalan & Bowen, 2020).
- Social and regulatory dimensions including public perceptions of restoration and adaptation interventions, strategies to involve Indigenous groups whose traditional sea Country estates include the GBR (hereafter referred to as “Reef Traditional Owners”) and stakeholders in research and decision‐making, the distribution of intervention benefits and risks, and policy reforms to support adaptation decision‐making (Shumway et al., 2023).

Future implementation of interventions developed through RRAP will require assessment of risks to the Reef's outstanding universal value (OUV) as inscribed in the GBR World Heritage Area. It will also require the involvement of Reef Traditional Owners, who have growing rights in the GBR, and evidence of consultation with stakeholders. Given the limited focus of the OUV on natural and aesthetic values, it is likely that more expansive consideration of social risks will be needed to secure and maintain political support. This article is an outcome of a conceptual project to inform RRAP's approach to risk characterization.

### 2.2 Research methods

The article is informed both by our reading of the literature and by an extensive program of social research—activities particularly germane to this article including:

- Face‐to‐face semistructured interviews with 140 members of the Reef community, including Traditional Owners, stakeholders, and managers, to explore the potential implications of novel technological interventions in reef management.
- Co‐development of a Biocultural Risk and Opportunity assessment framework to be used by Reef Traditional Owners to inform their decision‐making in the GBR. The framework is the result of a collaboration with the Indigenous Partnerships team of the Australian Institute of Marine Sciences, the Reef Restoration and Adaptation Science and Crown of Thorns Starfish Technical Working Group, and several Traditional Owner groups. Importantly, it may also be used by Reef Traditional Owners to identify the kinds of partnerships they would like to enter into for potential future deployment of technologies on their sea country.
- Stakeholder Advisory Group deliberations involving 11 GBR community members in discussions about the research and deployment of new reef restoration technologies in the GBR region.
- An expert elicitation workshop involving researchers from across RRAP tasked with identifying a comprehensive list of plausible outcomes associated with intervention along with what is known about potential impact pathways, distribution across communities, and possibilities for management.

Although we do not present comprehensive results, we draw on these activities throughout the article to illustrate theoretical and methodological issues associated with social risk in the context of assisted ecosystem adaptation. A summary of social risks identified through these activities is presented in Section 4.

### 2.3 The challenge assisted adaptation presents for conceptualizing risk

The scale and novelty of programs like RRAP raise a diversity of issues illustrative of the broader challenge assisted ecosystem adaptation presents for risk management. Noting that myriad definitions and metrics are available to operationalize the broad understanding of risk as the consequences of activities or processes for things people value (see Aven et al., 2018), we organize this overview of such challenges using the four types of “risk problem” identified by the International Risk Governance Council; that is, problem issues or scenarios characterized by simplicity, complexity, uncertainty, and/or ambiguity (see also Table 1) (Renn, 2005; see also Renn, 2015; Rosa et al., 2014). These, in turn, suggest options to operationalize risk management relevant to specific risk scenarios.

**TABLE 1:** Key concepts and definitions.

Risk problem characteristics	Simplicity	Risk problems characterized by well‐understood causal chains and predictable outcomes.
	Complexity	Risk problems characterized by multiple causal chains, intervening variables, and feedback loops; inhibiting prediction based on the behavior of one component.
	Uncertainty	Risk problems characterized by significant knowledge gaps, nonlinearity, and variability in how systems respond to the same stimuli.
	Ambiguity	Risk problems characterized by competing perspectives on their meaning (interpretive ambiguity) and/or moral implications (normative ambiguity).
Social risk types	Social risks	Risks to individuals or groups associated with social change triggered by the decisions of external actors.
	Biocultural risks	Risks to linguistic, cultural, and biological diversity, particularly as they relate to the rights and responsibilities of Indigenous peoples.
	Risk perceptions	Subjective judgment or appraisal of risk by individuals and groups.
	Business/project risks	Risks to the operation of a business or implementation of a project, particularly those that arise from interaction with stakeholders and communities.
	Social vulnerability	A lack of access to resources that, combined with exposure to potential hazards, places populations “at risk” of failure to withstand those hazards.
Other relevant concepts	Risk	The consequences of an activity or process for things that humans value.
	Risk governance	The totality of actors, rules, conventions, processes, and mechanisms involved in the characterization, management, and communication of risk.
	Social impacts	Any change, positive or negative, in the culture, livelihoods, and well‐being of individuals and groups that arises from the decisions of external actors.
	Social learning	Changes in how social groups understand problem contexts and/or the relationships between their individual and shared interests.
	Strengths‐based approaches	Focus on how knowledge, aspirations, and social relationships in communities often considered “at risk” can be harnessed to support autonomy, leadership, and capacity development.

*Simple* or linear risk problems can be managed using routine processes and instruments including technical standards and risk–benefit analysis (Renn, 2005). Ecosystem management options that can be assessed and controlled through these routine approaches can improve the effectiveness and acceptability of assisted adaptation by reducing cost, uncertainty, and perceptions of needless risk‐taking. Improving “conventional” management practices like pest control, for example, will reduce pressures on coral reefs and allow more time for evolutionary processes to build climate resilience (Anthony et al., 2020). In addition, as experience with restoration has grown, the Great Barrier Reef Marine Park Authority has taken steps to streamline approvals for “proven” low‐risk restoration methods including small‐scale outplanting of locally provenanced coral fragments at sites managed by tourism operators or Reef Traditional Owners (McLeod et al., 2022). Stewardship agreements for these sites will be used, in other words, as mechanisms to treat certain reef restoration practices as routine objects of risk management.

*Complex* risk problems are characterized by the interaction of multiple causal chains, intervening variables, and feedback loops, the management of which may require multidisciplinary inputs, systems modeling, and implementation of multiple safety systems (Aven et al., 2018; Renn, 2005). This is reflected in the urgency of helping reefs and other ecosystems adapt before they suffer irreversible climate damage through the implementation of multiple interventions at a variety of scales (Anthony et al., 2020; McLeod et al., 2019). Large‐scale coral aquaculture and automated outplanting using corals bred for heat resistance could be used, for example, to promote gene flow and accelerate adaptation. Ecosystem modeling, scenario analysis, and structured decision frameworks are used within RRAP to facilitate collaboration among ecologists, engineers, and social scientists and to inform decisions about investment in intervention research and development. Although models cannot be expected to capture the full ecological complexity of the world's largest coral ecosystem, it is their inability to help manage uncertainty and ambiguity that places larger constraints on risk analysis.

*Uncertainty* may reflect knowledge gaps, nonlinearity, variability in how people and systems respond to the same stimuli, and other challenges to modeling causal pathways and their consequences (Renn, 2005). Managing uncertainty, according to Renn (2005), may involve taking precautions (seeking to contain and minimize risk in response to characteristics such as persistence and irreversibility) or building resilience (the capacity to cope with and adapt to surprises). In assisted ecosystem adaptation, however, the application of either strategy is complicated by uncertainties regarding (1) the timing and magnitude of climate change; (2) the effects of climate change on ecosystem processes critical to adaptation; (3) the long‐term impacts of assisted adaptation measures initiated in anticipation of climate change; and (4) the long‐term impacts of pursuing the alternative, or counterfactual, scenario of no anticipatory action to assist ecosystem adaptation.

In RRAP, system modeling, scenario analysis, and structured decision frameworks are intended to help decision‐makers consider the implications of complexity and uncertainty. It must be acknowledged, nonetheless, that while some prospective interventions are designed to have limited spatial and temporal reach (e.g., SRM), others are intentionally irreversible (e.g., genetic selection), and all have the potential to generate unanticipated outcomes (Schipper, 2020; Silvestrini et al., 2015; Sovacool et al., 2023). When they cannot be resolved through research, these uncertainties require dialogue between experts, policymakers, stakeholders, and publics to evaluate trade‐offs between under‐ and overprotection against risk.

Dialogue must also confront *ambiguity* associated with competing perspectives on the severity of, and justification for, risk‐taking. Ambiguity can be interpretive (relating to the sense and meaning people make of risk and potential risk management options) or normative (relating to the moral evaluations people make of those same risks and strategies) (Aven et al., 2018; Renn, 2005). Both these dimensions are evident in the scientific literature on assisted adaptation and concomitant debate over the (1) feasibility of developing practical, cost‐effective, and timely interventions; (2) likelihood of developing consensus on adaptation given the polemicized state of climate politics; (3) suitability of governance regimes for managing adaptation interventions and their potential for unintended and cross‐boundary consequences; (4) ethics of intervention in ecosystems traditionally managed to conserve “natural” values; and (5) possibility of developing valid and reliable knowledge about as yet unrealized futures (Vella et al., 2021).

These last two points speak to the question of whose values and knowledge count in adaptation decision‐making. Ecosystem management based on Western conservation paradigms has a history of displacing Indigenous peoples from protected areas, ignoring interdependencies between biological and cultural diversity, and denying Indigenous knowledge of ecosystem function, change, and care (Fletcher et al., 2021). As Vella et al. (2021) argue, bringing Indigenous peoples’ values and knowledge into conversation with scientific perspectives is critical to understanding and resolving moral and interpretive ambiguities associated with assisted ecosystem adaptation in the GBR and elsewhere.

## 3 CONCEPTUALIZING SOCIAL IMPACTS AND RISKS

Although the benefits of improved ecosystem resilience for communities and industries may seem self‐evident, they can be complicated by uneven distribution, spatial and temporal distance between interventions and their effects, and myriad other sources of complexity, uncertainty, and ambiguity (Vella et al., 2021). Before considering the implications of assisted ecosystem adaptation for people in more detail, we review how the terms “social impact” and “social risk” are used in the literature and how the relationships between the two might be usefully conceptualized.

### 3.1 Social impacts

Social impacts were defined by the US Interorganizational Committee on Guidelines and Principles for Social Impact Assessment (SIA) as any change, positive or negative, in the ways “people live, work, play, relate to one another, organize to meet their needs, and generally cope as members of society” (Burdge et al., 1995, p. 11). The committee goes on to say this “includes cultural impacts involving changes to the norms, values and beliefs” through which people understand themselves and their communities.

The linkage of SIA to approval processes for infrastructure, resource development, and other projects has been associated, in practice, with a narrow focus on the prediction of social impacts with little subsequent monitoring and few opportunities for impacted stakeholders and community members to participate in decision‐making (Lockie et al., 2008). The project approval focus of SIA, and of environmental impact assessment more broadly, has led to the promotion of alternative approaches including strategic environmental assessment and cumulative impact assessment (CIA). These alternatives reflect the importance to sustainable development of understanding how the effects of multiple projects and decisions interact across space and time to attenuate, amplify, and/or create new impacts.

The prevailing view among SIA practitioners and researchers is that impact assessment should not be seen as a technocratic exercise in predicting social change in advance of development but as an ongoing participatory process of “analyzing, monitoring and managing the social consequences of planned interventions” (Esteves et al., 2012, p. 34). This emphasizes the importance of understanding the secondary and cumulative impacts that develop over the full life cycle of projects (Lockie et al., 2009; Taylor et al., 2003), of adapting to unexpected impacts (Kaplan‐Hallam & Bennett, 2018), and of undertaking assessments in decision environments based on collective learning and negotiation (Lockie, 2001; O'Faircheallaigh, 2010; Webler et al., 1995).

### 3.2 Social risk

Myriad definitions of “social risk” can be found in the literature addressing matters as diverse as the psychology of interpersonal interaction, the operation of insurance and capital markets, the causes of economic inequality and vulnerability, perceptions of technological hazards, potential sources of social and political conflict, possibilities of almost any kind of harm to people, and others. Since the early 2000s, the term has become popularized through the mining industry to denote the potential for conflict over the perceived social or environmental impacts of business activities to disrupt operations and increase costs (Kemp et al., 2016). This conception has been criticized both for its conflation of risks to business with risks to host communities and for prioritizing the former over the latter (Graetz & Franks, 2016; Kemp et al., 2016). Clarity over the use of this term is needed, not for the sake of choosing one definition of social risk over another, but for understanding the multiple dimensions of risk relevant in a given context and the relationships between each (Graetz & Franks, 2016).

According to Graetz and Franks (2016), “social risk” is used to denote: the risk of negative social outcomes arising from business or program activities; lay peoples’ perceptions of risk and the ways in which these diverge from expert assessments; risks to business activity or project implementation due to social and political disruption; and the identification of populations “at risk” or in need of social protection. All are potentially important to understanding the social dimensions and consequences of adaptation decision‐making and are defined in more detail below. In addition, we define biocultural risk, a concept identified by First Peoples with customary rights in the GBR as a priority for conceptual and methodological development and which highlights interdependencies between environment and culture and the imperative experienced by First Peoples to care for traditional estates (e.g., Brierley, 2020; Walker et al., 2019).

This article adopts the following terminology:

- *Social risks* are defined as risks to individuals and groups that arise as a consequence of social change precipitated by the decisions of external actors (Graetz & Franks, 2016). These may include risks to social and economic well‐being, culture, human rights, health and safety, and environmental quality. Further, social risks may be associated both with the direct or immediate consequences of intervention, and with the indirect (or second‐ and third‐order) consequences of long‐term social and environmental change initiated by intervention.
- *Biocultural risks* are defined as risks to biocultural diversity—that is, the dynamic interplay of linguistic, cultural, and biological variability generative of the full diversity of life (Maffi, 2005). Biocultural approaches to environmental management recognize the interdependence of biological and cultural diversity and the contribution Indigenous and local knowledges make to the resilience of human and nonhuman communities (Gavin et al., 2015). Biocultural approaches acknowledge the rights and responsibilities of all parties, especially those of Indigenous peoples who are custodians of much of the world's biodiversity but whose rights are often compromised by conventional approaches to conservation. Biocultural diversity is thus inseparable from rights to self‐determination, participation in decision‐making, access to resources and so on embedded in the UN Declaration on the Rights of Indigenous Peoples (UNDRIP) (Gavin et al., 2015). Although threats to these rights are examples of social risk, identifying them as biocultural risks stresses the unique consequences of ecosystem change for Indigenous peoples and the unique rights to exercise epistemic and political authority accorded to them in the UNDRIP.
- *Risk perceptions* are defined as understandings and interpretations of risks as shaped by traditional, local, and practical knowledge, as well as by values, trust in information sources, emotional and affective responses to change, communication channels, and other aspects of individual and collective subjectivity (see Maclean et al., 2021; Renn & Benighaus, 2013; Slovic, 2011). Risk perceptions, in turn, shape individual and collective responses to potential futures, altering behaviors and investments in ways that can amplify or attenuate the material likelihood and character of risk outcomes (Kasperson et al., 2022; Renn et al., 1992; Siegrist & Árvai, 2020). It follows that risk perceptions are not antithetical to objective calculations of risk but that the relationships between lay and expert knowledge are interdependent and recursive (Kasperson & Kasperson, 1996; Klinke & Renn, 2021).
- *Business (or project) risks* are defined as risks to the operation of a business or implementation of a project including, but not limited to, risks associated with finance and markets, regulatory issues, health and safety, reputation, and interaction with stakeholders and communities (Esteves et al., 2017). The risk of conflict with stakeholders and communities has helped popularize the idea in the resources and other sectors of a “social license to operate” (e.g., Luke et al., 2018; Prno, 2013) or “corporate social responsibility” (Wilburn & Wilburn, 2014). While it should not be assumed that an absence of conflict or negative risk perceptions among stakeholders and communities signifies acceptance of an operation or intervention, interest in social license does point to the potential for social concerns to spill over into increased project costs and scrutiny from regulators and investors. Such spillover may serve to mitigate the risk of negative impacts but it may also, importantly, undermine potential benefits associated with project implementation.
- *Social vulnerability* refers to a lack of access to resources that, together with exposure to potential hazards, places populations “at risk” (Adger & Kelly, 1999). Indicators of social vulnerability typically include a variety of spatial, economic, demographic, and other factors that serve as proxies for peoples’ capacity for coping or adapting in the face of adversity. As proxies, these indicators provide limited insight into how power and agency shape actual outcomes. Nonetheless, it remains important to recognize that adaptation interventions have the potential to reinforce, redistribute, and create new sources of vulnerability and, conversely, to build collective capacity for change (Eriksen et al., 2021; Jozaei et al., 2022). These potentials have a direct bearing on the magnitude and distribution of social risks as defined above (Esteves et al., 2017), particularly those associated with environmental changes extending beyond project timelines.

Risk perceptions, business risks, and social vulnerabilities interact and contribute to the potentialities we define as social and biocultural risks. Considering them together may contribute to what Kemp et al. (2016) refer to as a more social understanding of risk, one that is sensitive to how people experience and perceive threats and opportunities and how particular threats and opportunities come to matter differently across groups and contexts.

### 3.3 The relationship between social impacts and social risks

Early work in SIA treated risk as synonymous with increased exposure to industrial hazards such as pollution and accidents. Neither the US Guidelines and Principles for SIA (Burdge et al., 1995) nor subsequent best‐practice guidelines advocate risk assessment as a methodology for characterizing social impacts (Lockie, 2001; Vanclay, 2003). Although risk assessment has entered SIA through public and private sector social protection standards and social impact management plans (Franks & Vanclay, 2013), risk remains less studied than impacts in the SIA literature (Kemp et al., 2016).

The understanding of risk assessment as a calculus of probability and consequence is commonplace in the literature on social impacts and risks (Esteves et al., 2017). This positions social risks as expected or perceived threats associated with planned intervention, and social impacts as the material realization of those risks. Social impacts, in other words, are the material and cognitive consequences of risk‐taking and risk assessment is the process of estimating their likelihood (Esteves et al., 2017; Vanclay, 2002). As impacts can be positive and negative, it is implicit in this understanding that risk assessment should address the potential for failure to achieve positive outcomes alongside the potential to generate harm.

Mahmoudi et al. (2013, p. 3) offer a different perspective on the relationship between social impacts and risks, based on the proposition that “risk refers only to the uncertain social consequences” of an activity or event, whereas impacts are certain or expected. While risk is often equated with uncertainty (see Aven et al., 2018), uncertainty is also often theorized as the antithesis of risk (see Wong & Lockie, 2020). Zinn (2008, p. 10), for example, argues that the concept of risk “implies that an uncertain future can be made available to human action,” making risk assessment a practice that transforms uncertainty into an object of management, enabling action in circumstances that might otherwise be considered too dangerous or simply too poorly understood. As we argue in more depth below, rendering risk manageable may, or may not, require uncertainties to be resolved.

Despite the analytical distinction drawn here between risks and impacts, in practice, the two concepts are closely related. The anticipation of change may be sufficient, for example, to provoke immediate consequences ranging from anxiety and/or excitement over perceived threats and opportunities to education, career, and investment decisions, conflict over the desirability of change, shifting social and political alliances, and so on, all of which may change over the course of a project as particular outcomes are realized and expectations for the future change (Lockie, 2001). Further, the extended time horizons relevant to assisted ecosystem adaptation interventions support an integrated approach to the identification and mitigation of impacts alongside the characterization and management of risks (including risks of failure to achieve positive intended outcomes). While the concept of impact is arguably binary (they either are, or are not, realized), the concept of risk is temporally dynamic. It functions to make prospective change tangible and present and turns possibilities into objects of management.

**FIGURE 1:**
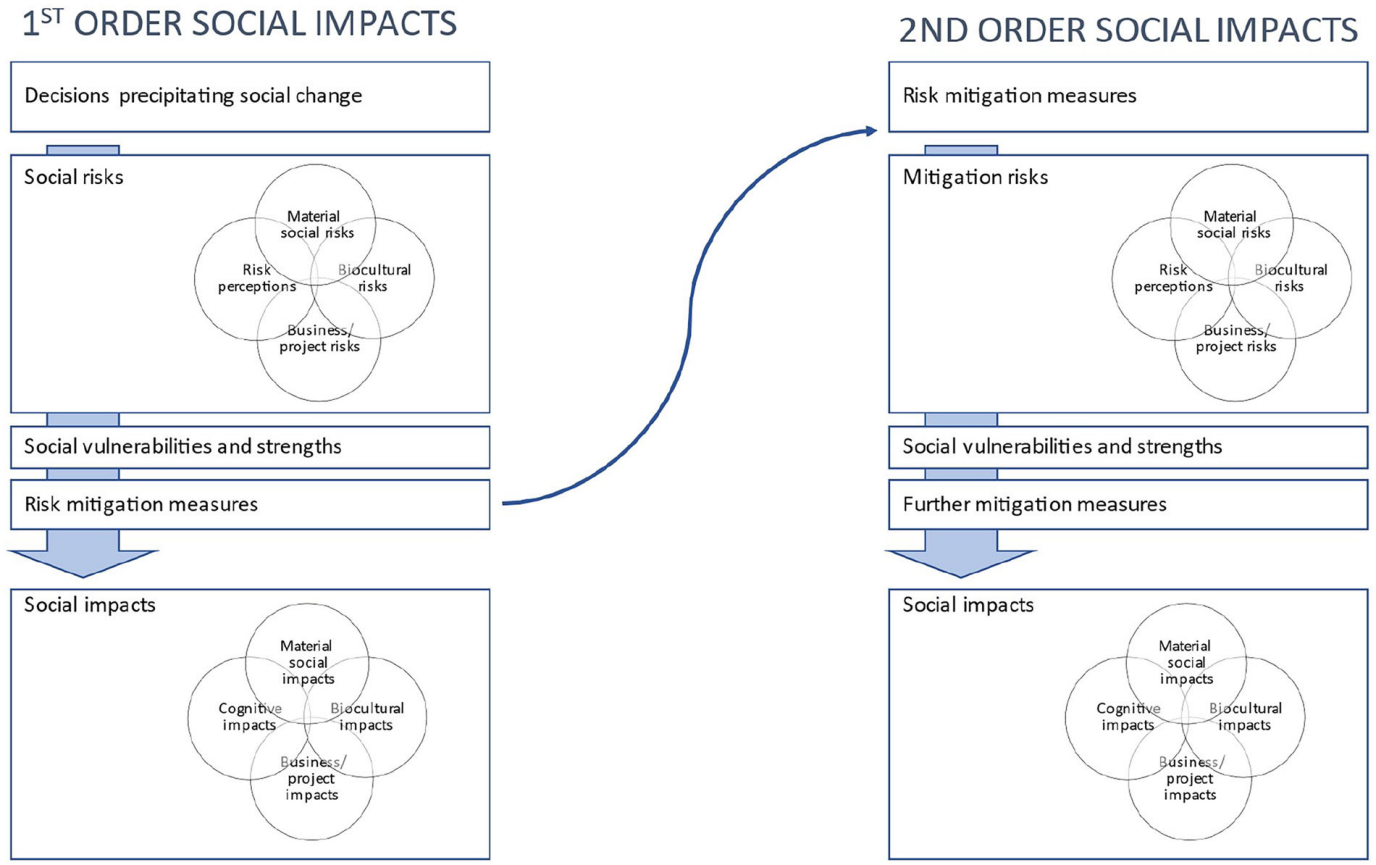
Relationships among multiple dimensions of social risk and impact.

Figure 1 illustrates relationships between social risks and impacts. It shows the:

- Iterative relationships between risk perceptions and material risks to livelihoods, well‐being, culture, and so on, biocultural risks and risks to business or project implementation;
- Role social vulnerability and active risk mitigation play moderating the translation of risks into impacts; and
- Importance of considering second‐ and third‐order risks associated with intervention to mitigate the direct impacts of social change.

### 3.4 Managing uncertainty and ambiguity through risk governance

According to Zinn (2016), adapting to global environmental change demands active decision‐making in the face of profound and likely enduring uncertainty. Decision‐makers find themselves caught between the risk‐minimizing norms of the precautionary principle and the risk‐taking ambition of changing the future through experimentation with innovative techniques of ecosystem design and reinstatement. This requires acting on a future that cannot be measured or observed (Rounsevell et al., 2021).

Not that the future is entirely opaque. A range of tools including forecasts, models, scenarios, and, of course, risk assessment exist to transform it into an object that can be managed (Lockie, 2014). The challenge is not that the consequences of assisted adaptation or other interventions cannot be known, but that some kinds of uncertainty are more amenable to resolution than others, and different kinds of risk demand different approaches to characterization and management.

Returning to the four types of “risk problem” identified by the International Risk Governance Council (i.e., simple, complex, uncertain, and/or ambiguous risk scenarios) (Renn, 2005), several points relating to the assessment and management of social risk bear drawing out. First, apart from distinctly linear and routine risks, all risk problems call for assessment that is sensitive to risk perceptions and consequently to societal responses to risk (Renn, 2005). Complex risk problems demand assessment that is inclusive of risk perceptions and exposures among affected stakeholders and that avoids untested assumptions about how people and institutions will behave in relation to risk and risk mitigation (Lockie & Wong, 2018). Second, risk problems characterized by high levels of uncertainty and/or ambiguity demand both an understanding of risk perceptions and exposures and increasing levels of stakeholder and public involvement in assessment and management (Renn, 2005). This evokes a third point, namely, the introduction of additional uncertainties associated with the unpredictable nature of decision‐making and limits to the authority and effectiveness of actors and governance systems (Rounsevell et al., 2021). This is of particular relevance to risk problems such as assisted ecosystem adaptation that cross jurisdictional boundaries, involve multiple stakeholders and governance actors with potentially competing interests, aspirations, and values, and are potentially vulnerable to politicization (Sovacool et al., 2024).

Risk problems may, of course, evince more than one risk characteristic. The purpose of highlighting the multiple characters of risk is not to imply that contemporaneously complex, uncertain, and ambiguous risks are impossibly hazardous but to identify appropriate approaches for their assessment and management. Risk governance is described by Klinke and Renn (2021) as a marriage between collective decision‐making and risk assessment. It embodies the “totality of actors, rules, conventions, processes, and mechanisms” through which “risk information is collected, analyzed and communicated” and through which risk “management decisions are taken” (Renn, 2005, p. 81). It includes government and private actors, formal and informal regulatory arrangements, and multiple forums for deliberation and negotiation open to a range of decision‐makers, experts, stakeholders, and publics. While risk governance is neither a silver bullet for the resolution of complexity, uncertainty, and ambiguity nor a fixed set of institutional arrangements for transforming them into objects of management, guiding principles for effective risk governance can be identified nonetheless (Klinke & Renn, 2021). The importance of three will be discussed here: social learning; politically legitimate decision‐making; and strengths‐based participation (see Table 2).

**TABLE 2:** Risk governance principles, types of risk, and their products.

Risk governance principles	Main risk type	Resolves or manages	Produces/promotes
Social learning	Risk perceptions Material social risk	Value conflicts Changing risk context	Adaptability
Legitimate decision‐making	Business/project risk	Accountability, responsibility, and fairness concerns Noncompliance	Institutional trust Collaboration
Strengths‐based participation	Biocultural risk Vulnerability	Diversity Inequality	Justice Interdependence Care

Social learning refers to changes in how both problem contexts and relationships between individual and shared interests are understood (Webler et al., 1995). It is as much about responsibility, moral reasoning, and solidarity as it is about knowledge, methods, and communication. Social learning can address the relationships identified in the previous section between *risk perceptions* and *social risk* outcomes and facilitate the resolution of value conflicts. Inclusive and continuous processes of social learning are needed to address the dynamic (Klinke & Renn, 2021) or mutable (Wong, 2015) nature of risk (that is, the propensity of risk to change through time and when examined from different perspectives) and thus to support adaptive, sustainable risk governance (Klinke & Renn, 2021). Such learning is enhanced, according to Gerlak and Heikkila (2011), when provision is made for: diverse sources of knowledge; decision‐making structures that are open to internal and external debate; actors with responsibility to link and coordinate across organizational boundaries; and experimentation in means of communication and interaction.

Transparency, communication, inclusion, and accountability provide foundations for trust, alignment between lay and expert risk perceptions and, ultimately, decisions being regarded by stakeholders and publics as fair and legitimate (Jijelava & Vanclay, 2017; Klinke & Renn, 2021; Wong, 2015). A lack of legitimacy in relation to decisions or the institutions responsible for risk governance can create *business or project risk*. It may also amplify risk by promoting noncompliance with control measures and/or by destabilizing decision‐making and increasing uncertainty (Rounsevell et al., 2021). Institutional arrangements, coupled with worldviews and historic access to political resources, can impact sense of place, connection to culture, well‐being, and recreational enjoyment. This again speaks to the fundamental interdependence of risk perceptions and material risk outcomes.

Interdependence is fundamental to *biocultural risk*. While it is important to ensure that risk decision‐making does not deepen social inequalities and *vulnerabilities*, many biocultural approaches advocate for a strengths‐based narrative driven by Indigenous care and responsibility for changing biocultural systems (e.g., Bullock et al., 2020; Lyons et al., 2020; Maclean et al., 2013; Nursey‐Bray et al., 2019). In part, this reflects the view that a strengths‐based approach, which recognizes the rights of Indigenous peoples and the need to contest ongoing processes of colonization, is the right thing to do (Bullock et al., 2020; Lyons et al., 2020; Nursey‐Bray et al., 2019). It also, however, acknowledges the diverse Indigenous governance systems that already structure relations between people and place (Artelle et al., 2019) across different Indigenous cultures and ways of relating to places and ecosystems (e.g., Rose, 1996; Parlee et al., 2005), and is critical to addressing vulnerability and designing robust systems for risk governance.

## 4 IMPACTS AND RISKS ARISING FROM ASSISTED ECOSYSTEM ADAPTATION

The degradation of coral reefs is associated with widespread and severe impacts on the livelihoods, culture, nutritional status, and psycho‐social well‐being of coastal communities and has become a focal point for significant conflict over societal responses to the threat of climate change. Persistent coral bleaching may, for example, reduce tourism income, provoke grief among residents, or disrupt cultural connections and sense of identity among Indigenous communities. In the framework presented in this article, these are treated as first‐order impacts, with the nature and magnitude of each moderated by an interplay between *social risk* (e.g., change in income from tourism); *risk perceptions* (e.g., decisions to visit or not visit the Reef based on awareness of coral bleaching); *vulnerability* (e.g., degree of exposure to declining visitation); and pre‐emptive action taken in relation to the hazard (e.g., adaptation interventions).

**FIGURE 2:**
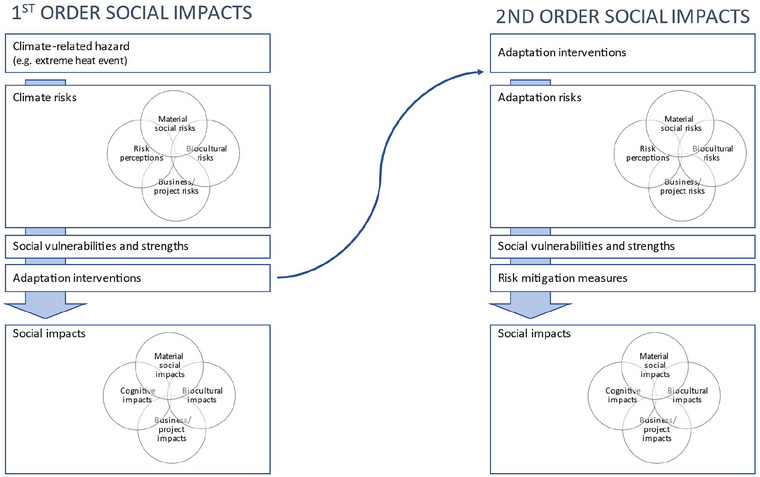
Relationships among social risks and impacts arising from assisted ecosystem adaptation.

Adaptation actions, such as large‐scale intervention to build climate resilience in coral reef ecosystems, generate their own social impacts. Figure 2 presents these as second‐order impacts. Some are simple while others are characterized by complexities, uncertainties, and ambiguities and are less easily resolved. Will ecosystems subject to assisted adaptation measures, for example, retain their desirability as tourism destinations? Will assisted adaptation be interpreted as an expression of responsibility and care or an exercise in artifice and despoilation?

**FIGURE 3:**
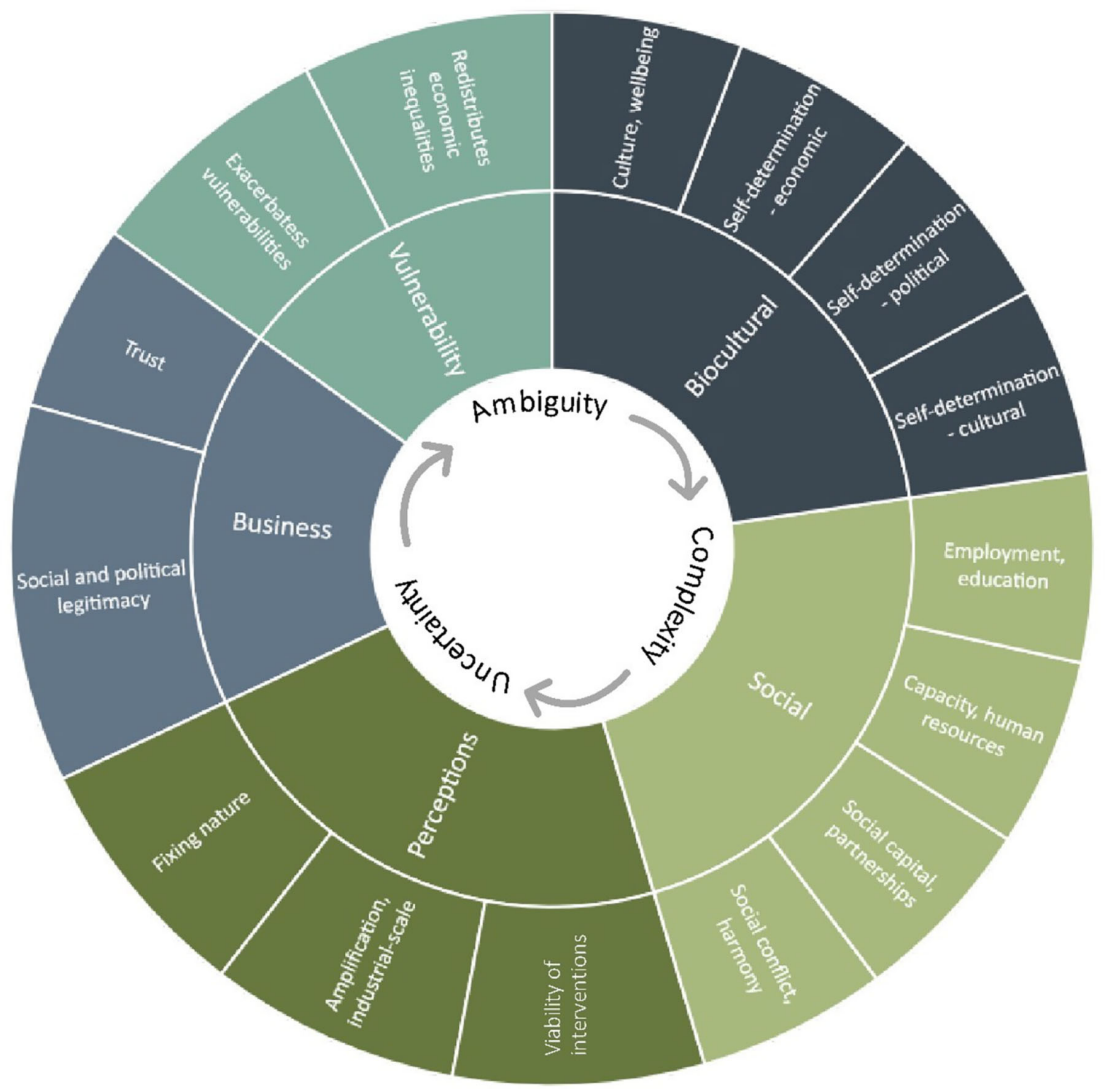
The social risk wheel illustrating five categories of social risk with examples.

Understanding risks arising from adaptation is useful for managing potential material, cognitive, and biocultural impacts over time. The social risk wheel (Figure 3) illustrates the types of risks associated with the reef adaptation interventions discussed in Section 2. Trust and acceptance of regulatory and scientific institutions, and the interventions themselves, can influence the emergence of business risks during planning or implementation. For example, tensions can emerge as governance models, partnerships, and funding are initiated. Material social impacts may include the diversification of income sources for tourism operators that undertake coral restoration or target new markets in restoration and adaptation eco‐tourism. Perception plays a large role in determining the magnitude and nature of any change in tourist visitation, as how individuals perceive adaptation interventions and expectations of nature itself will drive the desirability of the destination. The potential for marginalized communities to be excluded from participatory dialogues in adaptation planning can materialize into inequitable access to financial and vocational opportunities, thereby further reinforcing vulnerabilities (Pelling, 1998). This is especially true in technologically novel programs when participation is driven by preexisting social networks, equipment, financial and human resources, and technical expertise. In the biocultural domain, risks include conflicts with holistic approaches to caring for land and seascapes, cultural displacement and disempowerment, loss of autonomy over the sea country, and trauma related to colonial dispossession.

### 4.1 Risk governance and assisted ecosystem adaptation

Adaptation decision‐making is both a matter of scientific and technical concern and one of politics, values, and ethics. It speaks to the multiple ways in which people make sense of and respond to environmental change and of strategies enacted to mitigate and adapt to it. Risk governance that is inclusive of multiple perspectives and knowledges is needed to manage (not necessarily resolve) the uncertainties and moral and interpretive ambiguities embedded in assisted adaptation.

There is also a place here, we argue, for rigorous social research. Assessing the likelihood of social impacts is complicated by moral and interpretive ambiguity over the shift to active restoration and adaptation intervention in protected areas and uncertainty over how the intervention will be perceived by visitors, residents, and others. It is possible, nonetheless, to inform public deliberation on these issues through place‐focused analysis to identify who is likely to benefit from adaptation interventions and whether preexisting vulnerabilities might be exacerbated. Are benefits clustered around specific areas or do they favor individuals and organizations with preexisting social networks and the financial, human, and technical resources to successfully tender for work? Social learning (Table 2) that explores attachment to place and value systems can recommend appropriate governance models and structures for community participation in developing climate adaptation responses. For example, cultures with strong place‐attachment are likely to desire deeper involvement and greater levels of authority (Adger et al., 2013).

Choosing approaches to resolve or address risks complicated by moral and interpretive ambiguity requires a deep knowledge of the factors that interact with risks and amplify, accentuate, or mitigate impacts. Individual and collective perceptions of risk and levels of confidence are connected not only to the effectiveness of adaptation technologies but to “grassroots” social objectives and aspirations (e.g., employment benefits). Risks to the social and moral legitimacy (Table 2) of adaptation plans and actions arise from perceptions of their acceptability in the absence of meaningful community inclusion in defining objectives and aspirations for success (Schlosberg et al., 2017). A lack of public deliberation around what constitutes “good adaptation” can exacerbate inequitable participation processes in planning and implementation by enabling dominant views within funding and implementing organizations to frame the objectives of adaptation measures without reference to community aspirations (Eriksen et al., 2021).

Uncertainty can arise from perceptions of governance, decision‐making, and leadership in a large‐scale adaptation program. These perceptions are shaped not only by institutional arrangements but also by worldviews and historic patterns in access to political resources. For example, threats to self‐determination, participation in decision‐making, and access to resources can exacerbate the deeply embedded, transgenerational effects of colonization for Indigenous peoples, and the continued development by non‐indigenous people of ancestral territories (Bullock et al., 2020; Lyons et al., 2020; Nursey‐Bray et al., 2019). These biocultural risks are the obverse of strengths‐based participation (see Table 1). Australia's National Strategy for Just Adaptation (Future Earth Australia, 2022) strongly signals that processes to nurture and include the knowledge, needs, aspirations, and capabilities of all residents, particularly diverse groups, are needed to improve the collective national ability to adapt. To manage biocultural and vulnerability risks a strengths‐based and participatory approach that recognizes the full range of perspectives, including marginalized groups and communities, is important (Bennett, 2022).

## 5 CONCLUSION

While still prospective, the turn toward human‐assisted ecosystem adaptation exemplifies a broader shift in our understandings of “nature” and “nature protection” under climate change. Part of this, according to Zinn (2006), involves a societal recalibration of environmental decision‐making away from an emphasis on risk avoidance and minimization and toward bolder steps and greater risk‐taking. However, like any other climate adaptation strategy, risks associated with assisted ecosystem adaptation still require robust systems of management, including the early and considered evaluation of risks, costs, and benefits to rights holders, communities, and other stakeholders (Eriksen et al., 2021; Tedesco et al., 2023).

The turn toward assisted ecosystem adaptation also exemplifies the risk problems identified by the Risk Governance Council as demanding of multidisciplinary, participatory, and informed risk governance (Renn, 2005). The social risk framework presented here does not provide straightforward answers about the ethics, public acceptability, or social impacts of assisted adaptation because these play out through processes characterized by complexity, uncertainty, and ambiguity. It does draw attention, however, to the role of risk perceptions, social vulnerabilities and capacities, social risks, business risks, and biocultural risks in shaping assisted adaptation outcomes. Rigorous social research is thus as fundamental to characterizing and managing adaptation risk as any other science.

Our framework also highlights the importance of aligning risk governance processes with the principles of social learning, politically legitimate decision‐making, and strengths‐based participation. Risk governance is not a silver bullet for managing uncertainty and ambiguity but a means through which decision‐making can be imbued with transparency, communication, inclusion, accountability, trust, and fairness. It is a means for blurring the neat conceptual boundaries between social, technical, and ecological risk assessment—something most apparent in relation to biocultural risk which, as defined in this article, speaks to the epistemic authority of Indigenous peoples and to self‐determination, rights, and the material interdependence of linguistic, cultural and biological diversity.

This article is a response to the challenge of assisted ecosystem adaptation, but its framework is relevant to other social risk problems. Unpacking the complexity of risks posed by climate adaptation, and climate change more broadly, advances the development of impact assessment approaches for large and complex environmental programs. Despite the strong consensus from governments at all levels to commit to adaptation targets, the implementation of large‐scale adaptation in practice is lagging (O'Regan et al., 2021). Characterizing the risks posed by climate adaptation is foundational to assisted‐ecosystem adaptation more broadly, along with the mobilization of financial resources (Barr et al., 2021), a stronger evidence base of the feasibility and effectiveness of interventions (Singh et al., 2022) and capacity building within local communities and practitioners (Jijelava & Vanclay, 2017). At the same time, transformational adaptation strategies are needed that address the causes of social vulnerability and climate change (Fedele et al., 2019; Hellin et al., 2021).

## FUNDING INFORMATION

Financial support for this research was provided through the Reef Restoration and Adaptation Program, funded by the partnership between the Australian Government's Reef Trust and the Great Barrier Reef Foundation.

## CONFLICT OF INTEREST STATEMENT

The authors declare no competing interests.

## Data Availability

The data that support the findings of this study are not publicly available due to ethical restrictions.

## Footnotes

1 Partners include the Australian Institute of Marine Science, The Great Barrier Reef Foundation, CSIRO, Queensland University of Technology, James Cook University, the University of Queensland, and Southern Cross University.
